# Controlled therapeutic cholesterol delivery to cells for the proliferation and differentiation of keratinocytes†

**DOI:** 10.1039/d4tb01015a

**Published:** 2024-10-28

**Authors:** Krzysztof Berniak, Ahmadreza Moradi, Agata Lichawska-Cieslar, Weronika Szukala, Jolanta Jura, Urszula Stachewicz

**Affiliations:** a Faculty of Metals Engineering and Industrial Computer Science, AGH University of Science and Technology Krakow Poland ustachew@agh.edu.pl; b Department of General Biochemistry, Faculty of Biochemistry, Biophysics and Biotechnology, Jagiellonian University Krakow Poland; c Jagiellonian University, Doctoral School of Exact and Natural Sciences Krakow Poland

## Abstract

The challenge of enhancing wound healing and skin regeneration, particularly in conditions like burns and diabetic wounds, necessitates innovative solutions. Cholesterol, often associated with cardiovascular diseases, plays vital roles in cellular functions, maintaining skin integrity and preserving the skin barrier. Here, we explore cholesterol's significance, its influence on keratinocytes, and its potential application in skin regeneration. The study utilizes electrospun polyimide (PI) fibers as a cholesterol carrier model and investigates its impact on HaCaT keratinocytes, marking the first time tracked cholesterol delivery from the scaffold into cells. We demonstrate that an optimal concentration of 0.7 mM cholesterol in the medium enhances cell proliferation, while higher concentrations have negative effects. Cholesterol-enriched scaffolds significantly increase cell proliferation and replicative activity, especially in a 3D culture environment. Moreover, cholesterol influences keratinocyte differentiation, promoting early differentiation while inhibiting late differentiation. These findings suggest that cholesterol-loaded scaffolds can have applications in wound healing by promoting cell growth, regulating differentiation, and potentially accelerating wound closure. Further research in this area will lead to innovative wound management and tissue regeneration strategies.

## Abstract 2

The research concept involves investigating how cholesterol endocytosis impacts cells using electrospun fibers as a carrier. We studied keratinocyte proliferation and evaluated gene expression levels for markers of early and late differentiation.

## Graphical abstract

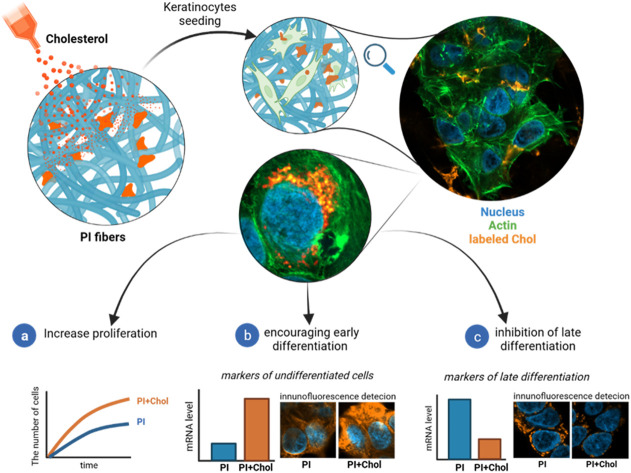

## 1. Introduction

The challenge that consistently exists in the field of improving the wound healing process and understanding skin regeneration in various conditions, such as burns or diabetic wounds, is the need to find new solutions to enhance these processes.^1–3^ One of the intriguing research directions is the role of certain lipid molecules, like cholesterol, in wound healing and skin regeneration processes. Why might cholesterol prove to be beneficial in these contexts? Despite often being linked to negative associations due to its role in cardiovascular diseases, cholesterol plays essential functions and is indispensable for the proper functioning of the body. This article aims to explore the significance of cholesterol, its effects on keratinocytes, and its participation in the skin regeneration and wound healing process.

Cholesterol plays several critical functions in the human body. It acts as a structural component of cell membranes, imparting integrity, and fluidity to these lipid bilayers.^4^ It also stabilizes cell membranes, preventing their breakdown under stressful or damaging conditions.^5^ Furthermore, cholesterol modulates the activity of membrane proteins, including receptors for hormones and neurotransmitters.^6^ Additionally, cholesterol is essential for the synthesis of steroid hormones, including cortisol, aldosterone, and sex hormones like estrogen and testosterone.^4,7^ Importantly, cholesterol acts as a signaling molecule in cells, influencing processes such as cell differentiation and proliferation.^8^ In addition to all that has been mentioned before, cholesterol is crucial for maintaining the integrity and barrier function of the skin.^9,10^ Its role is related to the regulation of differentiation and proliferation of keratinocytes, which are cells found in the epidermis, the outermost layer of the skin.^11,12^ Therefore, cholesterol contributes to the formation and restoration of the protective skin barrier. Cholesterol also influences the permeability of the epidermis, ensuring the selective movement of molecules and preventing excessive water loss.^13^ In addition to its various beneficial roles, cholesterol has emerged as a key player in the field of lipid nanoparticles, particularly in the formulation of effective liposomal systems for cellular delivery. Cholesterol also plays a crucial role in stabilizing liposomal membranes, enhancing their biocompatibility, and facilitating efficient delivery of therapeutic agents to target cells. Studies have shown that cholesterol-based gemini cationic lipids significantly improve gene transfection efficiency and serum compatibility. Cholesterol is essential for forming stable liposomal structures, which effectively encapsulate and deliver DNA to cells.^14^ Furthermore, the development of a cationic cholesterol-based nanocarrier for gene delivery to cancer cells demonstrated high transfection efficiency and significant induction of apoptosis in cancer cells, with cholesterol being crucial for achieving efficient gene delivery both *in vitro* and *in vivo*.^15^ Additionally, research highlighting the importance of lipid interactions in membrane stability and function is essential for designing cholesterol-containing liposomal systems.^16^ Insights into molecular stability and reactivity further support the design and optimization of cholesterol-based lipid nanoparticles.^17^

Despite studies showing the multifaceted role of cholesterol in cells, it is not usually used as a standalone pharmaceutical treatment for skin conditions. However, cholesterol plays a role in the composition of the skin and is an essential component of the stratum corneum, the outermost layer of the skin. The stratum corneum helps maintain the skin's barrier function, preventing moisture loss and protecting against external irritants.^18–20^ In some pharmaceutical and skincare products, cholesterol may be included as an ingredient to help restore or support the skin's natural barrier function.^21–23^ These products are often formulated to address conditions like dry or damaged skin, eczema, or certain dermatological conditions. Cholesterol may be combined with other ingredients like ceramides and fatty acids to create a more comprehensive barrier-repairing effect.^21^

In the present investigation, we employed electrospun polymer fibers as a cholesterol carrier model. Previous literature has documented the successful utilization of alternative biocompatible polymers as dressings and drug carriers, including oils for treating skin ailments like atopic dermatitis.^24–26^ Recent works have shown that integrating antibacterial drugs with polymers significantly improves antibacterial potency against pathogenic bacterial strains. For example, films made from polyvinyl alcohol (PVA) modified with organic acids like lactic acid and malic acid exhibit much better antibacterial properties, making them useful for food packaging applications.^27^ Additionally, studies on polymeric nanoparticles have demonstrated that conjugating antibiotics like gentamicin with polymer nanoparticles greatly enhances antibacterial efficacy. These nanoparticles facilitate precise drug delivery to infection sites, boosting therapeutic effectiveness.^28^ Moreover, polymer-antibiotic conjugates show improved antibacterial activity due to the synergistic effects of polymers and drugs. Research on polymer-based drug carriers, such as those using poly(vinyl alcohol), indicates that combining these polymers with antibiotics significantly increases their bactericidal capabilities.^29^ Furthermore, polymeric drug carriers have been shown to prevent bacterial adhesion and colonization effectively. For instance, functionalized carbon dot polymers display enhanced antibacterial properties due to their unique surface structures and ability to generate reactive oxygen species.^30^ Lastly, another study demonstrated that integrating antimicrobial drugs with biocompatible polymers like chitosan resulted in significantly enhanced antibacterial properties against various bacterial strains. This was particularly evident in their effectiveness in wound healing applications.^31^ These patches not only regulate drug release properties but also provide physical and mechanical protection to the damaged skin area, exhibit a high surface area for gas exchange, absorb exudate, and are compatible with cells while being biodegradable.^32^ The utilization of biodegradable dressings composed of materials that undergo breakdown offers supplementary benefits of a non-toxic temporary solution.^33^ These dressings are frequently derived from plant-based or bacterial sources, which pose reduced environmental harm compared to conventional dressings from synthetic materials. Consequently, such dressings hold the potential to diminish medical waste, as they can naturally decompose after usage without necessitating specialized disposal storage.^34–37^

**Fig. 1:**
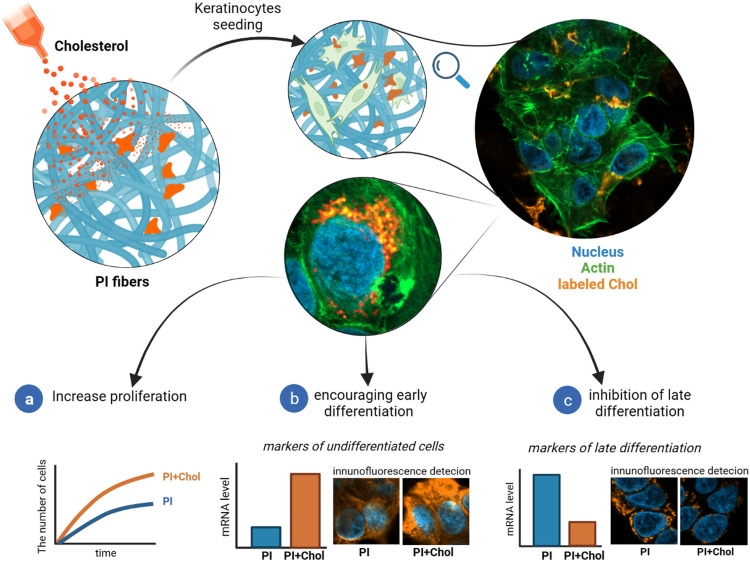
Conceptual representation of the research. The presented study elucidated the influence of cholesterol endocytosis using electrospun fibers on cells. The polyimide (PI) was used for the electrospun fibers. We examined the effectiveness of keratinocyte proliferation (a) and assessed the gene expression levels and markers of early (b) and late (c) differentiation.

This study explores the multifaceted functions of cholesterol in keratinocytes, highlighting its influence on proliferation, differentiation, and its potential application in skin tissue regeneration (Fig. 1). We track cholesterol uptake by cells to investigate its effects on the HaCaT cell line, a well-established model for studying keratinocyte biology, offering potential avenues for the development of advanced wound dressings and regenerative therapies.

## 2. Results and discussion

### 2.1. The impact of cholesterol on the keratinocyte cell culture

**Fig. 2:**
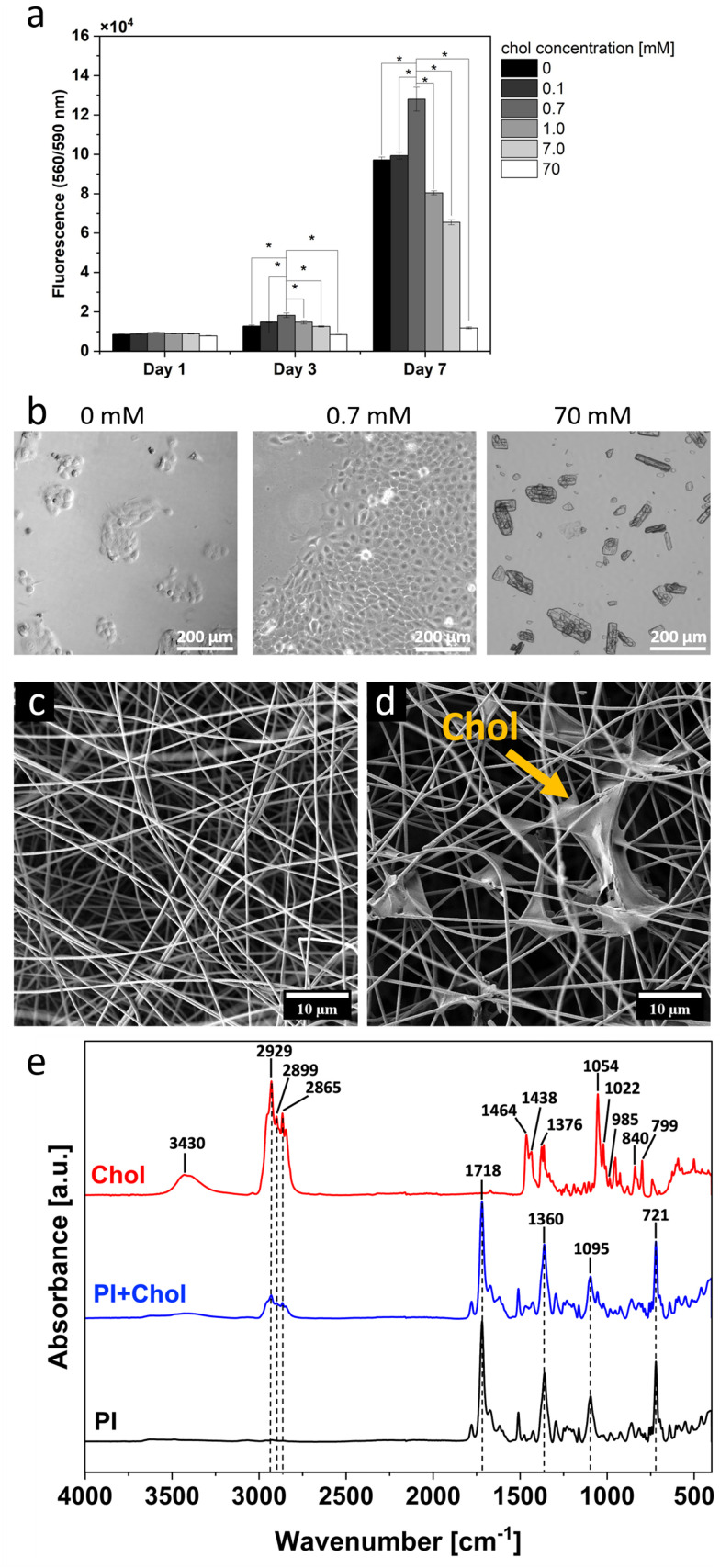
Manufacture of cholesterol-enriched fibers. (a) Proliferation assays of the keratinocyte HaCaT cell line in different cholesterol concentrations in medium. (b) Wide-field microscopic images of HaCaT cells after 7 days of cell culture in medium with additional 0, 0.7, and 70 mM of Chol, respectively. (c) SEM micrographs of PI fibers and (d) PI fibers with cholesterol. (e) FTIR results of both PI and PI + Chol scaffolds, and pure cholesterol. *Statistical significance calculated using ANOVA, followed by Tukey's *post hoc* test, *p* < 0.05; error bars are based on standard deviation.

The addition of varying quantities of cholesterol to the medium results in divergent, and sometimes even opposing, effects on cell proliferation and viability in the cell culture. Thus, we monitored the behavior of keratinocytes and their proliferation in cell cultures with different contents of cholesterol in the culture medium during 7 days (Fig. 2a). After one day, no significant differences in cell numbers were observed across the different concentrations. However, after 3 days of culture, the situation was drastically changed, and a significant increase in cell numbers was observed in the medium containing 0.7 mM cholesterol compared to the other cholesterol concentrations. The greatest difference in cell numbers was observed after 7 days of culture. Importantly, the culture with 0.7 mM cholesterol showed over 30% higher signal compared to the control culture without cholesterol. However, the higher concentrations of cholesterol had a negative impact on cell growth in the culture. A concentration of 1 mM cholesterol resulted in a decrease in cell numbers compared to the control, and 70 mM cholesterol was toxic to the cells, with a signal approximately 87% lower than the control culture. Images in transmitted light reveal the difference in cell count in media with varying cholesterol content (Fig. 2b). In the control culture (0 mM), numerous cell colonies consisting of several to several dozen cells are visible. At a cholesterol concentration of 0.7 mM in the medium, a significant increase in cell growth is observed compared to the control. In cultures with a cholesterol content of 70 mM, there are very few cells. However, cholesterol crystals are visible in the medium. Indeed, these results indicate that 0.7 mM cholesterol has the best positive effect on the cell proliferation rate.

### 2.2. Fiber characteristics

To produce a cholesterol-enriched polymer scaffold (PI + Chol), we used an electrospinning technique with simultaneous electrospray of a cholesterol solution with a final concentration of 0.7 mM, which achieved the best cell proliferation rate in the previous experiment (Fig. 2a). Considering the application of electrospun mats, we selected polyimide (PI), a robust material known for its thermal stability, chemical resistivity and excellent mechanical properties, including high tensile strength, toughness, and elastic modulus.^23,38,39^ Polymer scaffolds enable the cultivation of 3D cell cultures. This method of culture better replicates the structure of living tissues and allows for more realistic studies of cell interactions, including their interaction with the microenvironment.^40–42^ Studies have shown that electrospun mats possess the 3D structure, as demonstrated by previous studies using advanced imaging techniques like focus ion beam and scanning electron microscopy (FIB-SEM), revealing their complex architecture and cell infiltration into the electrospun scaffolds.^43–45^ Therefore, in the study, PI scaffolds without the addition of cholesterol were used as a control to minimize the influence of the 3D culture itself on the obtained results.^14^ Examination of SEM micrographs revealed no substantial disparities in the fiber morphology (Fig. 2c and d). However, SEM images (Fig. 2d) clearly demonstrated cholesterol accumulation within the interstitial spaces between fibers. The presence of cholesterol in the dressing was further confirmed by Fourier-transform infrared spectroscopy (FTIR) analysis (see Fig. 2e). The characteristic peaks of cholesterol are clearly visible in the obtained spectrum, as the broad band at 3430 cm^−1^ is attributed to O–H stretching. The peaks between 2800 and 3000 cm^−1^ show symmetric and asymmetric stretching vibrations, and the peaks at 1464, 1438, and 1376 cm^−1^ are related to the bending vibrations of the CH_2_ and CH_3_ groups. The sharp peak at 1055 cm^−1^ can be due to cholesterol ring deformation. The absorptions at 1022, 985, and 799 cm^−1^ are the result of aromatic substitution patterns, and the peak at 840 cm^−1^ shows the C–C–C stretching of the cholesterol molecule.^46,47^ The PI and PI + Chol scaffolds illustrate the characteristic absorptions of PI. The absorption bands at 721 cm^−1^ and 1095 cm^−1^ indicate imide ring deformation and OC–N–CO, respectively. The C–N stretch band was observed at 1360 cm^−1^, and the peak at 1718 cm^−1^ demonstrates C=O stretch.^48–50^ Moreover, as a result of adding the cholesterol to PI scaffolds, the new peaks at 2929, 2899, and 2865 cm^−1^ appeared in the PI + Chol spectrum, proving the presence of cholesterol in the electrospun scaffold.

### 2.3. Endocytosis of cholesterol into keratinocytes

**Fig. 3:**
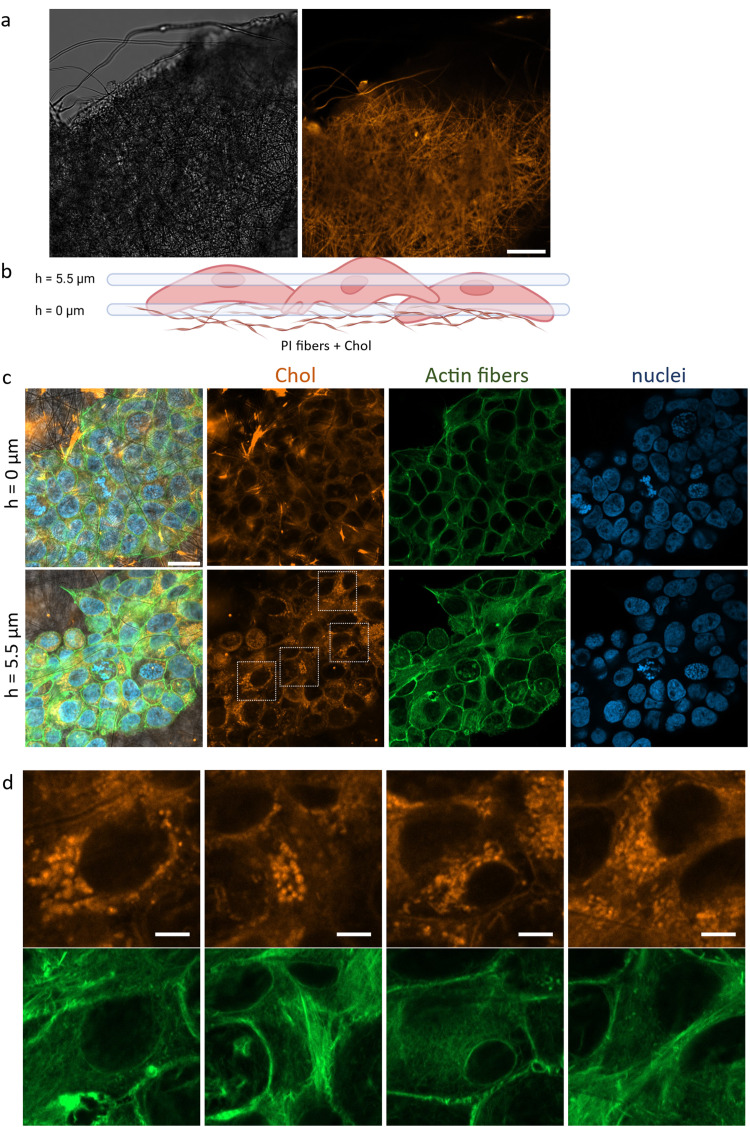
Endocytosis of labeled cholesterol from fibers into the cell. (a) Fibrous scaffold image in the transmitted light (grey) and CLSM image of labeled cholesterol (CholEsteryl BODIPY) on the PI scaffold. (b) The schematic illustrating the imaging approach at 2 focusing depths. (c) CLSM image of HaCaT cells on the PI scaffold with labeled cholesterol after 3 days of culture, showing cells on two heights. (d) Enlargement of areas in cells where cholesterol accumulates inside them. Labeled cholesterol ester (orange) by CholEsteryl BODIPY, actin fibers were stained with Alexa Fluor 488 Phalloidin (green), and nuclei were counterstained with DAPI (blue). (a) – Scale bar: 20 μm, (c) and (d): Scale bar: 5 μm.

Polymer scaffolds are an effective carrier of cholesterol to cells in their close vicinity. To detect cholesterol molecules from the scaffold that had entered the cells, we used fluorescently labeled cholesterol ester (CholEsteryl BODIPY) that was incorporated into the polymer fibers building the scaffold (Fig. 3a). To visualize the cells growing on the 3D polymer scaffold, we used a confocal microscope with which we took a series of images at different heights (Fig. 3b). After 3 days of keratinocytes culture, a significant signal from the labeled cholesterol inside cells was observed, indicating that cells growing on the scaffold absorbed cholesterol *via* endocytosis from their surroundings (Fig. 3c). Cells grown in the presence of the scaffold with added cholesterol, though not in direct contact with fibers, also absorbed labeled cholesterol (Fig. S1, ESI†), albeit at a much lower intensity than cells grown directly on the scaffold with labeled cholesterol. These findings suggest that cholesterol is slowly released from the scaffolds, even after 3 days of culture. This observation aligns with the proposed mechanism of drug release from electrospun fibers, where in stage 2, slow release controlled by diffusion occurs through the interconnected membrane structure.^51^Fig. 3c presents two specific planes wherein confocal microscopy captured images of cells cultivated on a cholesterol-enriched scaffold. The reference plane, denoted as *h* = 0, signifies the interface between the cells and the substrate. In the top left corner of the image, a pronounced orange signal emanates from labeled cholesterol deposited on the scaffold fibers. The second section (*h* = 5.5 μm) intersects the cell nucleus and demonstrates the aggregation of cholesterol molecules surrounding the nuclei. Subsequently, chosen magnifications of the imaged region (Fig. 3d) provide a detailed depiction of the precise pinpointing of cholesterol accumulations within the cells. Cholesterol accumulation around the cell nucleus can serve various functions, including influencing the organization of the cell membrane and the structures of proteins involved in regulating intracellular processes.^52,53^ Cholesterol aggregation around the nucleus can impact nuclear functions, such as gene expression and cellular signaling.^54,55^

### 2.4. Cholesterol increases cell proliferation on the 3D scaffold

**Fig. 4:**
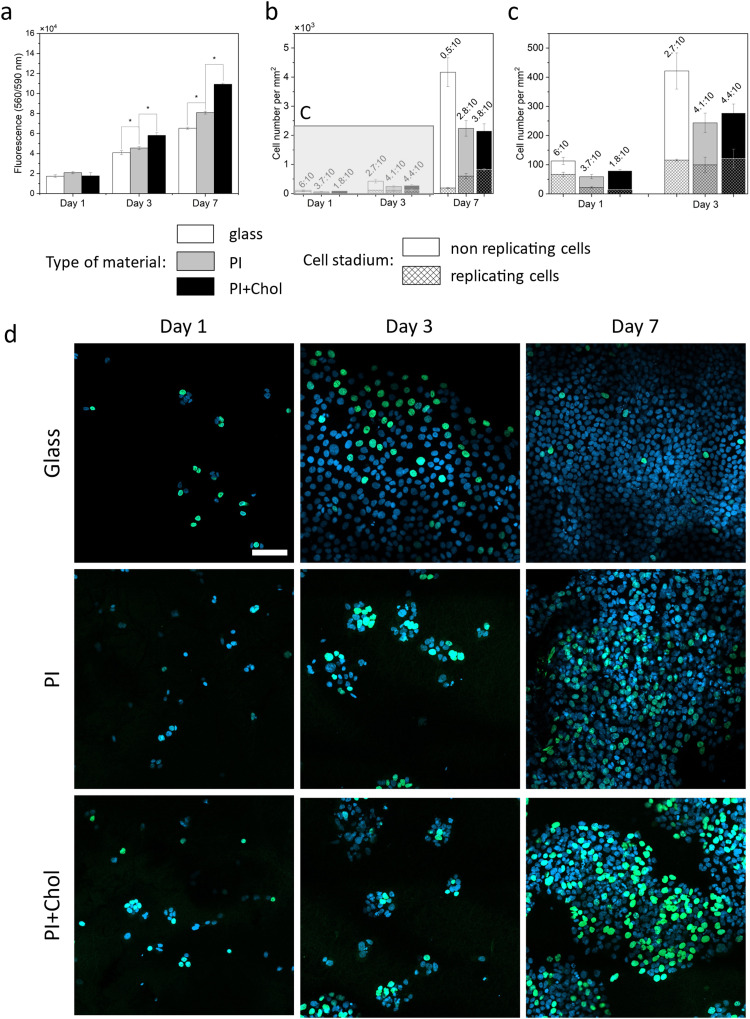
Analysis of cell replication activity. (a) Proliferation assays of keratinocytes’ HaCaT cells on PI fibers, PI + Chol, and glass as the control. (b) Number of keratinocytes, indicating replicating cells on PI and PI + Chol and glass slides after 1, 3, and 7 days of cell culture. The whole column represents the total number of cells. (c) Zoom in for B. (d) CLSM micrographs representing HaCaT cells on a glass slide, PI scaffolds, and PI + Chol scaffolds. Nuclei stained with DAPI (blue), incorporated EdU during replication stained with Alexa Fluor™ 488 (green). Scale bar = 100 μm. *Statistical significance calculated with ANOVA, followed by Tukey's *post hoc* test, *p* < 0.05; error bars are based on standard deviation.

Cholesterol-enriched scaffolds increase the rate of cell proliferation. We knew from previous experiments that the addition of cholesterol to the medium increases the rate of cell proliferation in 2D culture and keratinocytes cultured on the 3D scaffold absorb cholesterol molecules available on the surface of the scaffold fibers. The 3D scaffold causes changes in the rate of cell proliferation. The space for cells to grow is larger, making cells more willing to divide than in 2D culture. Thus, the next key step was to estimate the rate of cell proliferation on cholesterol-enriched scaffolds and also, as a control, on polymer scaffolds without added cholesterol (Fig. 4a). Notably, cells cultivated on cholesterol-enriched scaffolds exhibited significantly enhanced growth compared to cells on cholesterol-free scaffolds.

Following a 1-day incubation period, signal intensities were comparable across the examined samples. Subsequently, after 3 days, cell populations on PI + Chol fibers were 27% and 42% greater in number than those on the PI scaffold without cholesterol and glass substrates, respectively. After 7 days, cells growing on the PI scaffold with cholesterol demonstrated a remarkable increase in cell density, with cell numbers being 37% and 67% higher than cells on PI fibers and glass, respectively. The results confirm previous scientific reports indicating the lack of a toxic effect of PI in the form of fibers or film on cells.^56,57^ Furthermore, the enrichment of PI fibers with cholesterol led to an increase in the proliferation rate that had not been observed before in PI scaffolds.

Moreover, subsequent to a 7-day period of cultivation, keratinocytes were subjected to imaging analysis on PI and PI + Chol scaffolds, as illustrated in Fig. S2 (ESI†). Noteworthy, no discernible variations in cellular morphology were detected between the two experimental conditions. The cells exhibited a tendency to aggregate into colonies comprising numerous cells, exhibiting elongation along the fibers and generating multiple filopodia. This observation serves as compelling evidence, further substantiating the considerable biocompatibility of the investigated materials.

### 2.5. Cholesterol increases replicative activity

Cholesterol enhances cell replication, which we tested in cell cultures on both 3D scaffolds. (Fig. 4b–d). The increase in the number of cells growing on the cholesterol-enriched polymer scaffold compared to the scaffold alone showed the quantitative positive effect of cholesterol. In further studies, we focused on estimating the number of single cells showing replicative activity, as well as their localization relative to the other cells. Cholesterol enhances cell replication, as we have tested. The increase in the number of cells growing on the cholesterol-enriched polymer scaffold compared to the scaffold alone demonstrates the quantitative positive effect of cholesterol. In subsequent studies, our focus was on estimating the number of individual cells exhibiting replicative activity and their spatial distribution relative to the other cells. Additionally, we conducted an experiment on glass to illustrate that in a 2D culture, cells quickly occupy the entire available surface area, leading to a significant reduction in their replicative activity. The assay for replication activity used a labeled thymidine analog embedded in a newly synthesized DNA strand. All cell nuclei appear blue, while the green staining represents a labeled analog embedded in the DNA strand during the replication process. The number of cells observed on PI and PI with the added cholesterol scaffold is smaller than those on the glass due to the imaging technique and sample characteristics. On glass, cells grow in a flat manner, while scaffolds create a 3D space, resulting in the visualization of only a portion of the cells in a given area on confocal images. However, the ratio of green to blue signal cells remained constant regardless of the visible population. The replicative activity was initially 59% higher on glass compared to scaffolds. After 3 days, the efficiency of cell replication on glass began to decline, while on fibers, cell replication continued to increase (more than 40% in comparison to glass). After 7 days, cell proliferation on glass was limited, with only a small percentage of cells undergoing replication. In contrast, most cells replicated on the material containing cholesterol (almost 40%).

### 2.6. The effect of cholesterol on keratinocyte differentiation

The process of keratinocyte differentiation involves the gradual transformation of stem or progenitor cells into mature keratinocytes, which constitute the primary component of the epidermis. As cells differentiate, there are changes in their morphology, function, and gene expression, leading to the formation of epidermal cells capable of playing a protective role and forming the skin barrier.^58–60^ The efficiency of keratinocyte differentiation is altered by the presence of cholesterol available on the polymeric scaffold. To investigate this crucial process, we assessed the expression levels of widely recognized proliferation markers, early and late keratinocyte differentiation markers, as well as control markers for the inflammatory state. In our study, we focused on comparing the effects of polymer scaffolds with and without cholesterol on keratinocyte differentiation. While previous studies included a control group of cells grown on glass surfaces to establish baseline cellular responses, this specific analysis did not include the glass control as the primary objective was to investigate the differential effects of cholesterol-enriched and non-enriched 3D polymer scaffolds.

**Fig. 5:**
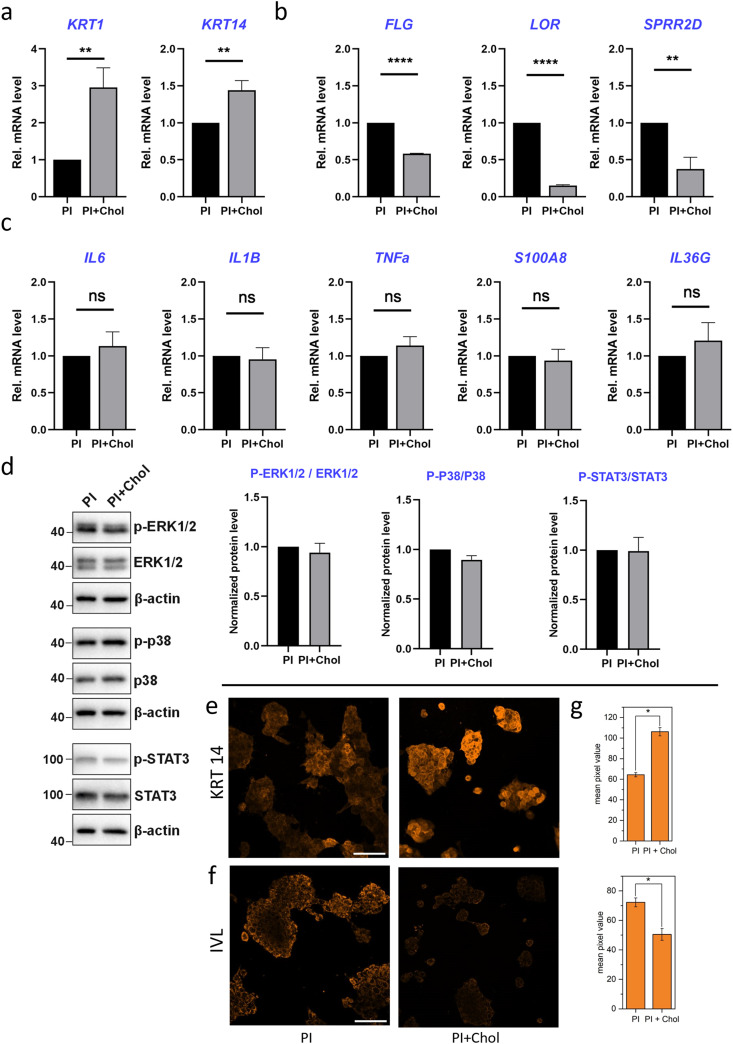
Analysis of expression levels of selected genes. (a)–(c) Quantitative PCR analysis of KRT14, KRT10, FLG, SPRR2D, IL6, IL1B, TNFa, S100A8, and IL36G expression levels in HaCaT cells grown on PI and cholesterol-enriched PI fibers for 5 days. (d) Representative western blot analysis and densitometric quantification of p-ERK1/2, p-p38 and p-STAT3 levels. Data are shown as mean and standard error of the mean, **p < 0.01, ****p < 0.0001 by an unpaired *t*-test. (e) and (f) CLSM micrographs representing KRT14 and IVL distributions in HaCaT cells grown on PI and PI + Chol, respectively. (g) Mean pixel intensity values from immunofluorescently labeled proteins KRT14 and IVL, respectively. KRT 14 and IVL were labeled by antibodies conjugated with Alexa 555 (orange). Scale bar: 100 μm. *Statistical significance calculated with ANOVA, followed by Tukey's *post hoc* test, *p* < 0.05; error bars are based on standard deviation.

Keratin-14 (KRT14) and keratin-1 (KRT1), well-known markers of early keratinocyte differentiation, were first analyzed. KRT14 is predominantly expressed in the basal layer, while KRT1 expression becomes prominent as cells progress toward the suprabasal layers.^61^ The sequential expression of KRT14 and KRT1 reflects the progression of keratinocytes through the early stages of differentiation. Enrichment of PI with cholesterol led to an increased transcriptional expression of proliferation and early differentiation markers: keratin-14 (KRT14) and KRT1, respectively (Fig. 5a). On the other hand, the level of mRNAs encoding terminal differentiation markers: filaggrin (FLG), loricrin (LOR) and small proline-rich protein 2D (SPRR2D) significantly decreased (Fig. 5b). FLG is involved in the formation of the skin barrier by promoting the aggregation of keratin intermediate filaments. LOR contributes to the structural integrity of the cornified envelope, while SPRR2D participates in the cross-linking of proteins during terminal differentiation.^62^ In Jans's publication, cholesterol depletion leads to a decrease in the expression of KRT14 and KRT1 while increasing late differentiation markers.^63^ We observe a similar trend in our results. HaCaT cells, a well-established model for studying keratinocyte biology, exhibit a proinflammatory phenotype under certain conditions. Among the pro-inflammatory genes, the expression of interleukin-6 (IL-6), IL1-β, IL-36γ, tumor necrosis factor α (TNF-α), and the antimicrobial peptide S100A8 is of particular interest.^64^ These genes are involved in immune responses and inflammation, which play critical roles in skin homeostasis and various dermatological disorders. Quantitative PCR analysis indicated that the expression of pro-inflammatory genes was not altered in HaCaT cells grown on the PI + Chol scaffold compared to the control PI scaffold. The transcripts encoding selected interleukin-6 (IL-6), IL1-β, IL-36γ, tumor necrosis factor α (TNF-α) and antimicrobial peptide S100A8 were unchanged (Fig. 5c). The last aspect that was analyzed was STAT-3 and MAPK signaling pathways. They have been implicated in regulating keratinocyte differentiation and gene expression in HaCaT cells. Activation of STAT-3 promotes keratinocyte proliferation, survival, and immune responses, while the MAPK pathway influences cell growth, differentiation, and inflammation.^65^ In keratinocytes, the MAPK pathway is activated by various extracellular stimuli, such as growth factors, cytokines, and environmental stressors. Upon activation, the pathway initiates a cascade of phosphorylation events, involving several kinases including ERK1/2 and p38.^66^ Western blot analysis indicated that STAT-3 and MAPK signaling pathways were not activated, confirming that the inflammatory response is not triggered by the PI scaffold (Fig. 5d).

To confirm the PCR results indicating elevated levels of proliferation and early differentiation markers in keratinocytes growing on PI fibers enriched with cholesterol, immunofluorescently labeled imaging of KRT 14 and involucrin (INV) proteins was performed (Fig. 5e and f). The same imaging parameters were used to record images for each kind of protein in cells on both types of scaffold. Assuming that the signal intensity is correlated with the amount of protein in the cell, the results obtained are consistent with the quantitative results obtained from the PCR method (Fig. 5c). Cells growing on the cholesterol-enriched scaffold show an increased protein level of KRT10 compared to the regular PI scaffold. The next protein, involucrin (IVL) is the marker of the late stages of differentiation and assembly of the cell envelope, which is a key structural component of corneocytes, the mature, toughened cells that make up the outermost layer of the epidermis.^67,68^ In cells growing on the PI + Chol scaffold, we have a decrease in the amount of IVL compared to the cells growing on PI fibers.

Our investigation into gene expression revealed that culturing cells on scaffolds enriched with cholesterol did not induce any additional inflammatory response in the cells. Conversely, we observed an elevated level of gene expression associated with the early differentiation of keratinocytes. Promoting early differentiation of keratinocytes can be advantageous, especially in scenarios like wound healing where rapid formation of a protective skin layer is desired.^69,70^

Finally, we observed a significant inhibition of the late differentiation process in keratinocytes growing on cholesterol-enriched polymer fibers. In the context of wound healing, especially in cases of burns or skin injuries, this is typically an undesired characteristic. However, delayed late differentiation can result in a prolonged proliferative phase, which might initially seem beneficial for covering the wound quickly. It is crucial to achieve a balance between proliferation and differentiation to ensure the formation of a functional epidermis.^71^ While our study shows that cholesterol-enriched scaffolds promote early differentiation and proliferation, the inhibition of late differentiation could mean that the final maturation and formation of the stratum corneum are delayed.

Despite this inhibition, the cholesterol-enriched scaffold system still provides significant advantages in the early stages of wound healing. The rapid proliferation and early differentiation of keratinocytes help to quickly form a protective layer over the wound, reducing the risk of infection and further injury. This initial response is crucial in acute wound management, providing immediate coverage and reducing healing time.

The inhibition of late differentiation might initially lead to an incomplete formation of the stratum corneum. However, the wound healing process is dynamic, and over time, the cells will likely progress to full differentiation once the immediate need for rapid coverage is met. The modulation of keratinocyte differentiation by cholesterol-enriched scaffolds could be fine-tuned in future studies to optimize both the early and late stages of wound healing.

The stratum corneum is essential for the barrier function of the skin. While our study observed a delay in the late differentiation process, it did not completely inhibit it. Over time, the differentiation process will continue, and the stratum corneum will form, although possibly at a slower rate.^72^ This delay can be advantageous in chronic wounds or conditions where excessive keratinocyte differentiation leads to pathological conditions such as hyperkeratosis.^73,74^

## 3. Conclusion

In our study, we applied PI (polyimide) fibers as a potential carrier for cholesterol in the wound healing process, representing a novel approach in utilizing polymer fibers as a scaffold for delivering bioactive molecules such as cholesterol. We primarily focused on the initial stages, involving the cellular response to cholesterol-enriched fibers. Crucially, by employing fluorescence labeling of cholesterol, we were able to monitor how HaCaT cells, serving as a keratinocyte model, received and incorporated cholesterol from the scaffold substrate. Labeled external cholesterol not only embeds itself in the cell membrane but also forms aggregates around the nucleus. Our findings revealed a new cellular response of keratinocytes to cholesterol-enriched polymer scaffolds. Not all cholesterol concentrations had positive effects. We demonstrated that different concentrations induce diverse and opposing biological responses. The optimal cholesterol concentration for promoting cell growth was 0.7 mM. Our results uncovered the molecular mechanism of keratinocyte responses to cholesterol, enhancing replicative activity and proliferation. This increased proliferation, crucial for wound healing, helps replace lost or damaged skin cells, thereby expediting the healing process. We revealed that culturing cells on cholesterol-enriched scaffolds did not induce additional inflammatory responses but did elevate gene expression associated with early keratinocyte differentiation. This is advantageous for wound healing as it promotes rapid formation of a protective layer. However, we observed significant inhibition of late differentiation, which can delay the maturation and formation of the stratum corneum. Despite this, the rapid proliferation and early differentiation of keratinocytes help quickly cover wounds, reducing infection risk and healing time. Over time, the cells will likely progress to full differentiation, ensuring the eventual formation of the stratum corneum. This delay can be beneficial for chronic wounds or conditions like hyperkeratosis, where excessive differentiation is problematic.^74^

These observations suggest the potential application of cholesterol-loaded fibers in the wound healing process. Further research and exploration of cholesterol's effects on wound healing mechanisms will pave the way for innovative strategies in wound management and tissue regeneration.

## 4. Experimental methods

### 4.1. Electrospinning

The electrospun scaffolds were prepared based on polyimide (PI, Ensinger Sintimid GmbH, Austria) solely and PI with cholesterol (Acros Organics, The Netherlands). PI granules were dried for 4 h at 50 °C (Drying Oven, POL-ECO Aparatura, Poland) prior to solution preparation, and the solutions were prepared by dissolving 18 wt% of PI in the dimethylacetamide (DMAc) (Avantor, Poland) and dimethyl sulfoxide (DMSO) (Avantor, Poland) mixture with a 7 : 3 mass ratio. The PI scaffolds were produced using an electrospinning machine (IME Technologies, The Netherlands) equipped with a climate control chamber, at RH = 60% and *T* = 24 °C by electrospinning for 1.5 h. The rest of the electrospinning parameters were: 18 kV of applied voltage, a solution flow rate of 0.30 mL h^−1^, and a nozzle-to-collector distance of 15 cm. A 21 gauge stainless-steel needle was used as the nozzle. Cholesterol solutions with 5 wt% concentration were prepared in DMAc. The concentration of cholesterol in the sprayed solution was chosen so that the amount of cholesterol deposited on a single PI scaffold after the addition of 1 mL of medium gave a target concentration of 0.7 mM. Scaffolds containing PI and cholesterol (PI + Chol) were produced by simultaneous electrospinning and electrospraying of PI and cholesterol solutions, respectively, using the two-nozzle setup. The parameters for electrospraying were: a nozzle-to-collector distance of 15 cm and a solution flow rate of 0.80 mL h^−1^. The rest of the parameters were kept the same as the electrospinning of PI. To obtain an even distribution of PI and cholesterol, both nozzles moved in reciprocating motions with 20 mm s^−1^ velocity along a 15 cm distance, and the collector rotated at a velocity of 10 rpm. Furthermore, scaffolds containing the orange fluorescent BODIPY FL cholesteryl ester (C12680, Thermo Fisher Scientific, USA), which can be used as a tracer of cholesterol, were prepared by adding the BODIPY FL cholesteryl ester solution in DMSO (0.05 wt%) to the cholesterol solution in a 1 : 7 mass ratio, using the same electrospraying parameters as the cholesterol solution.

### 4.2. Scanning electron microscopy (SEM)

The surface morphology of the scaffolds was analyzed *via* scanning electron microscopy (SEM, Merlin Gemini II, ZEISS, Germany). The samples were coated with an 8 nm layer of Au (sputter coater Q150RS, Quorum Technologies, UK), and the imaging was carried out at an accelerating voltage of 2.5 kV and a working distance of 4–5 mm using an SE detector.

### 4.3. Fourier transform infrared (FTIR) spectroscopy

Attenuated Total Reflectance–Fourier Transform Infrared Spectroscopy (ATR-FTIR, Nicolet™ iS™ 5, Thermo Fisher Scientific, USA) with the diamond crystal was utilized to identify the chemical composition of the cholesterol and the produced scaffolds. For each sample, the spectra were averaged over 64 scans in the range of 400–4000 cm^−1^, with a resolution of 4 cm^−1^. The obtained data were first normalized, and then the baseline was created and subtracted from the spectra using normalize and Peak Analyzer functions, respectively, in OriginPro software.

### 4.4. Cell culture and proliferation assay

The study utilized human keratinocytes-like cells (HaCaT) sourced from Sigma-Aldrich, UK. These cells were cultivated on polyimide (PI) fibers, as well as PI fibers with cholesterol and tissue culture polystyrene (TCPS) as a control. The culture was maintained at 37 °C and 90% humidity in a 5% CO_2_ atmosphere. The initial cell density was maintained at 2 × 10^4^ per sample. The HaCaT cells were grown in a complete culture medium comprising Dulbecco's modified Eagle medium (DMEM) supplemented with 10% fetal bovine serum (FBS), 2% antibiotics (penicillin–streptomycin), 1% l-glutamine solution, and 1% amino acids (Mem nonessential amino acid solution 100×), all of which were procured from Sigma-Aldrich, USA.

Cell proliferation was assessed using CellTiter Blue (Promega, USA) in HaCaT cell culture in two separate experiments. In the first experiment, HaCaT cells were cultured in culture medium with cholesterol, the final concentrations of which were 0.1 mM, 0.7 mM, 1.0 mM, 7.0 mM, and 70 mM, respectively. In the second experiment, HaCaT cells were cultured on PI, PI + Chol fibers and TCPS. Both experiments were conducted for 1, 3, and 7 days following the same protocol. For each time point, two replicates per sample type were used. At the end of each time point, the culture medium was removed, and 80 μL of CellTiter Blue reagent and 400 μL of fresh medium were added. The mixture was then incubated at 37 °C with 5% CO_2_ in a humidified atmosphere for 4 hours. Subsequently, 100 μL of the reaction solution was transferred to a new 96-well plate in four repetitions, and fluorescence was measured at an excitation wavelength of 560 nm and an emission wavelength of 590 nm using the microplate reader GloMax® Discover System (Promega, United States).

### 4.5. Replication test

HaCaT cells were grown on PI fibers and a glass slide (*Ø* 11 mm, Menzel-Glaser, Germany) as a control sample for 1, 3, and 7 days. The cells were then treated with 10 μM 5-ethynyl-2′-deoxyuridine (EdU) for 1 h. Afterward, the samples were washed with PBS and fixed with 4% paraformaldehyde, followed by permeabilization and blocking in 0.1% Triton X-100 (Sigma-Aldrich, USA) and 3% bovine serum albumin (Sigma-Aldrich, USA) at 25 °C, respectively. EdU incorporation was detected using the ClickiT™ EdU AF488 imaging kit (Invitrogen/Molecular Probes, USA). The cells were counterstained with 4′,6-diamidino-2-phenylindole (DAPI, Sigma-Aldrich, UK) for 15 min. The samples were imaged using a Zeiss LSM 900 confocal microscope (CLSM, Carl Zeiss Microscopy GmbH) with excitation at 405 nm and 488 nm laser lines and emission detection bands of 410–500 nm for DAPI and 500–700 nm for Alexa Fluor 488 coupled with incorporated EdU. The images were acquired using ZEN 3.1 software (Carl Zeiss Microscopy GmbH) and processed using ImageJ. A Plan-Apochromat 20 ×/0.8 M27 objective with a pixel size of 0.124 μm was used for imaging. The average density of replicated cells and all cells per mm^2^ was quantified from at least ten random fields (1 × 1 mm) for each sample using ImageJ 1.53v.

### 4.6. Cells morphology – imaging

Microscopic images of HaCaT cells after 7 days of cell culture in medium with added cholesterol were obtained using a wide-field microscope with a 20 ×/0.30 PH1 objective (Leica DMI1, Germany)

Confocal laser scanning microscopy (CLSM) was used for imaging the labeled cholesterol esters on PI scaffolds and estimating the cell morphology in culture. In this experiment, a multi-well plate setup was used where a coverslip was placed in each well, and polymer scaffolds cut to the appropriate diameter were placed on the coverslip. A suspension of cells was then seeded into this setup. The majority of the cells adhered to the scaffold fibers, while their small number settled on the glass coverslip. The entire process of fixation and staining of the cells was carried out within the multi-well plate. For imaging using confocal microscopy, the polymer scaffold along with the coverslip was transferred to a microscope slide. This setup allowed simultaneous imaging of cells in direct contact with the fibers and those adherent to the glass but growing in the presence of the polymer scaffold. The procedure for preparing samples for imaging was as follows. After 1, 3, and 7 days of cell growth, the samples were fixed with 4% paraformaldehyde (Sigma-Aldrich, United Kingdom) for 30 min. Subsequently, the samples were incubated in 0.1% Triton X-100 for 5 min, followed by washing in PBS and next incubated in 3% bovine serum albumin (BSA, Sigma-Aldrich, United Kingdom) for 1 h. The cells were incubated for 1 h in Alexa Fluor 488 Phalloidin (1 : 400, Thermo Fisher Scientific, United States) and then washed three times using PBS. Nuclear DNA was stained with DAPI (1 : 1000) for 5 min. The samples were mounted with Vectashield anti-fade mounting media (Merck, USA). The images were acquired using a confocal microscope (Zeiss LSM 900 Airyscan2 with ZEN 3.1 software, Germany). The following microscopy parameters were used: Plan-Apochromat 63 ×/1.4 Oil DIC M27, excitation using 405 nm, 488 nm and 561 nm diode lasers, emission detection bands of 400–490 nm for DAPI, 500–550 nm for Alexa Fluor 488 coupled with Phalloidin and 550–700 nm for CholEsteryl BODIPY. Registration was performed in sequential mode with a 16-bit dynamic range.

### 4.7. CLSM image signal intensity analysis

The staining protocol and imaging procedure were conducted in a similar manner as described in Chapter 5.6 “Cells morphology – imaging.” The experiment was conducted after 5 days of cell culture. To achieve greater imaging depth, a ×10/0.45 objective lens was employed, with registration parameters kept uniform for each protein across both scaffold types. For the labeling of KRT 14 and INL, mouse anti-cytokeratin 14 antibody (ab7800, Abcam) and rabbit anti-involucrin antibody (ab53112, Abcam) were employed, respectively. Secondary antibodies utilized were goat anti-rabbit secondary antibody conjugated with Alexa Fluor 555 (A2148, Thermo Fisher) and goat anti-mouse secondary antibody conjugated with Alexa Fluor Plus 555 (A727, Thermo Fisher). To estimate the mean pixel intensity value per cell, image analysis was performed. For each image, a cell area mask was generated, excluding the nucleus-occupied region. Pixels were selected from the raw microscopic image based on the mask and their mean value was calculated. The analysis was conducted using the ImageJ software.

### 4.8. RNA isolation and quantitative real-time PCR

For RNA isolation, the HaCaT cells growing on PI and PI + Chol fibers for 5 day were washed twice with cold PBS and lysed (together with scaffold) in Fenozol (A&A Biotechnology, Gdansk, Polska) and frozen at −20 °C. Then RNA was isolated using the standard phenol-chloroform method. The RNA concentration was determined using a NanoDrop 2000 (Thermo Fisher Scientific) and 1 μg of RNA was used to synthesize complementary DNA (cDNA) using M-MLV reverse transcriptase (Promega, Madison, WI, USA). Then, the 5-times diluted cDNA was amplified with SYBR Green Master Mix (A&A Biotechnology) and specific primers (Sigma-Aldrich) using a QuantStudio3 thermocycler (Thermo Fisher Scientific). Elongation factor 2 (EF2) was used as a reference gene. The following gene-specific primer pairs were used: for EF2 GACATCACCAAGGGTGTGCAG and TTCAGCACACTGGCATAGAGGC; for KRT1 ATTTCTGAGCTGAATCGTGTGATC and CTTGGCATCCTTGAGGGCATT; for KRT14 CCAGCTCAGCATGAAAGCATC and TGAGATCCAGAGGAGAACTG; for SPRR2D TGCATCTTCTCACCAAAGCCT and ACAGCTGAGGACTTCCTTTTCTT; for FLG AAGGTTCACATTTATTGCCAAA and GGATTTGCCGAAATTCCTTT; for LOR CCAGGGTGCCACGGAGGCGAAGGA and TGAGGCACTGGGGTTGGGAGGTAG; for IL6 GTGAAAGCAGCAAAGAGGCA and TCACCAGGCAAGTCTCCTCA; for IL1B TGGGTAATTTTTGGGATCTACACTCT and AATCTGTACCTGTCCTGCGTGTT; for TNFA CAGGCGGTGCTTGTTCCTCAG and GGGCTACAGGCTTGTCACTCG; for S100A8 GCTGGAGAAAGCCTTGAACTC and CCACGCCCATCTTTATCACCA; for IL36G GAAGGTTGGAGAACAGCCCA and GGTCCTACCAGTCTTGGCAC.

### 4.9. Western blot analysis

For protein isolation, HaCaT cells were washed twice with cold PBS and harvested (together with scaffold) in RIPA buffer supplemented with protease and phosphatase inhibitors (Roche). Then, lysates were centrifuged for 20 minutes at 12 000 × *g* at −4 °C and only supernatant was collected. The protein concentration was measured using a bicinchoninic acid assay. The cell lysates were separated by SDS-PAGE on 8% polyacrylamide gels and transferred to PVDF membranes (Millipore, Billerica, MA, USA). Densitometric quantification was performed using ImageLab (Bio-Rad, Hercules, CA, USA). Protein levels were normalized to the expression level of β-actin. The following antibodies were used in this study: mouse anti-β-actin (A1978; 1 : 2000; Sigma-Aldrich), rabbit anti-phospo-Thr202/Tyr204-ERK1/2 (4370, 1 : 2000, Cell Signaling Technology), rabbit anti-ERK1/2 (9102, 1 : 1000, Cell Signaling Technology), rabbit anti-phospo-Thr180/Tyr182-p38 (4511, 1 : 1000, Cell Signaling Technology), rabbit anti-p38 (9212, 1 : 1000, Cell Signaling Technology), rabbit phospo-Tyr705-STAT3 (9145; 1 : 2000; Cell Signaling Technology, Danvers, MA, USA), rabbit STAT3 (4904; 1 : 2000; Cell Signaling Technology), goat peroxidase-conjugated anti-rabbit (7074; 1 : 3000; Cell Signaling Technology) and goat peroxidase-conjugated anti-mouse (554002; 1 : 20000; BD Pharmingen).

## Data availability

The raw/processed data required to reproduce this finding are available on request from the authors.

## Conflicts of interest

The authors declare no conflict of interest.

## Supplementary Material

TB-012-D4TB01015A-s001
