# Supplementary material for: Controlled therapeutic cholesterol delivery to cells for the proliferation and differentiation of keratinocytes
Source: J Mater Chem B. 2024 Oct 28;12(43):11110–22. doi: 10.1039/d4tb01015a (PMC11515930; doi:10.1039/d4tb01015a)
Supplement: TB-012-D4TB01015A-s001 [file TB-012-D4TB01015A-s001.pdf]

Supplementary Information (SI) for Journal of Materials Chemistry B.
This journal is © The Royal Society of Chemistry 2024

Supporting Information

Controlled therapeutic cholesterol delivery to cells for proliferation and differentiation of keratinocytes

*Krzysztof Berniak, Ahmadreza Moradi, Agata Lichawska-Cieślar, Weronika Szukala, Jolanta Jura, Urszula Stachewicz**

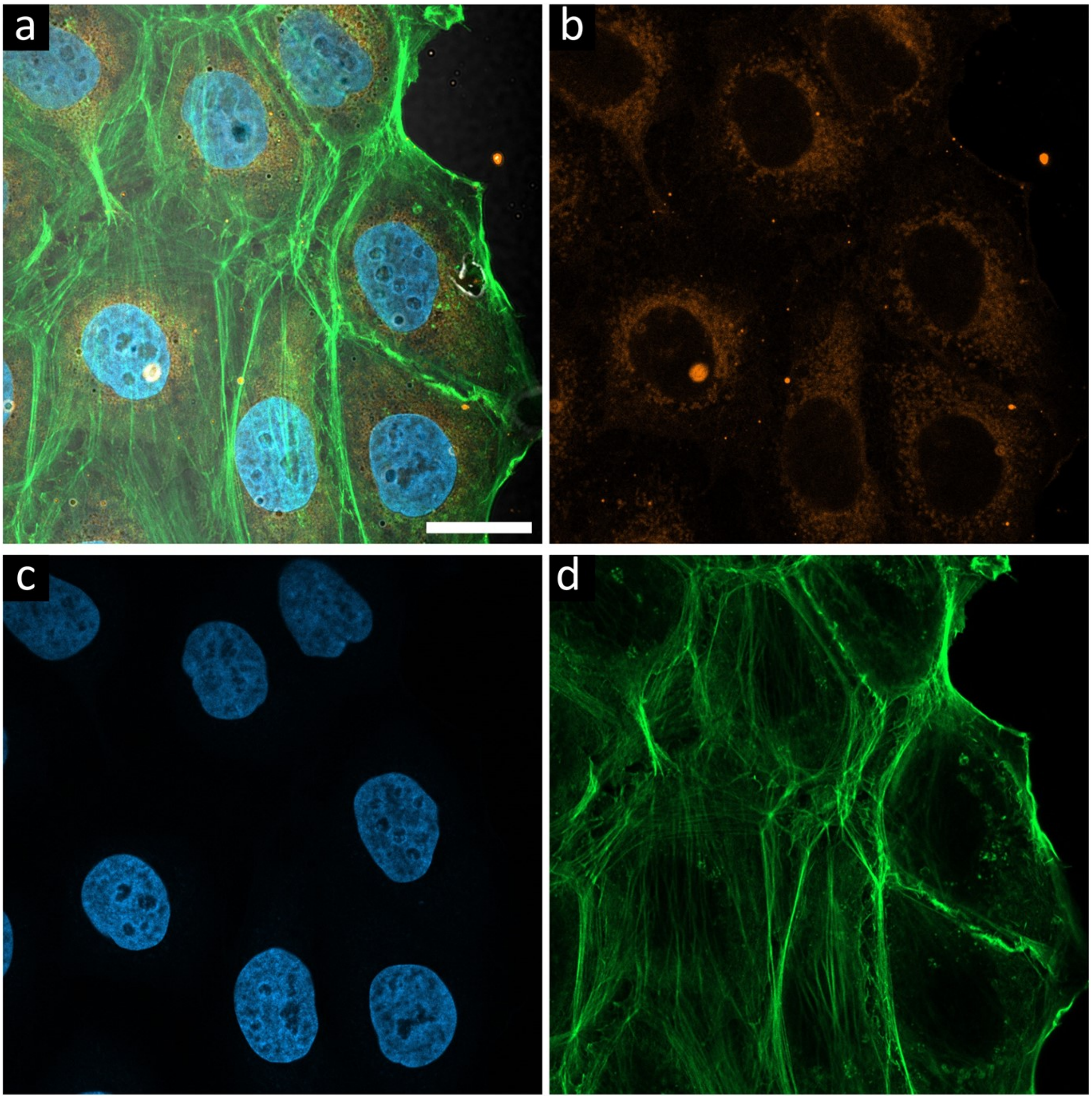

Fig. S1. Accumulation of labeled cholesterol in cells not having direct contact with the scaffold. a CLSM images of HaCaT cells on glass in the presence of a PI scaffold with added

cholesterol, after 5 days of culture. **b** Labeled cholesterol (orange) stained by CholEsteryl BODIPY**;** **c** the nuclei were stained with DAPI (blue), and **d** the actin filaments with Alexa Fluor 488 Phalloidin (green). Scale bar = 20 µm.

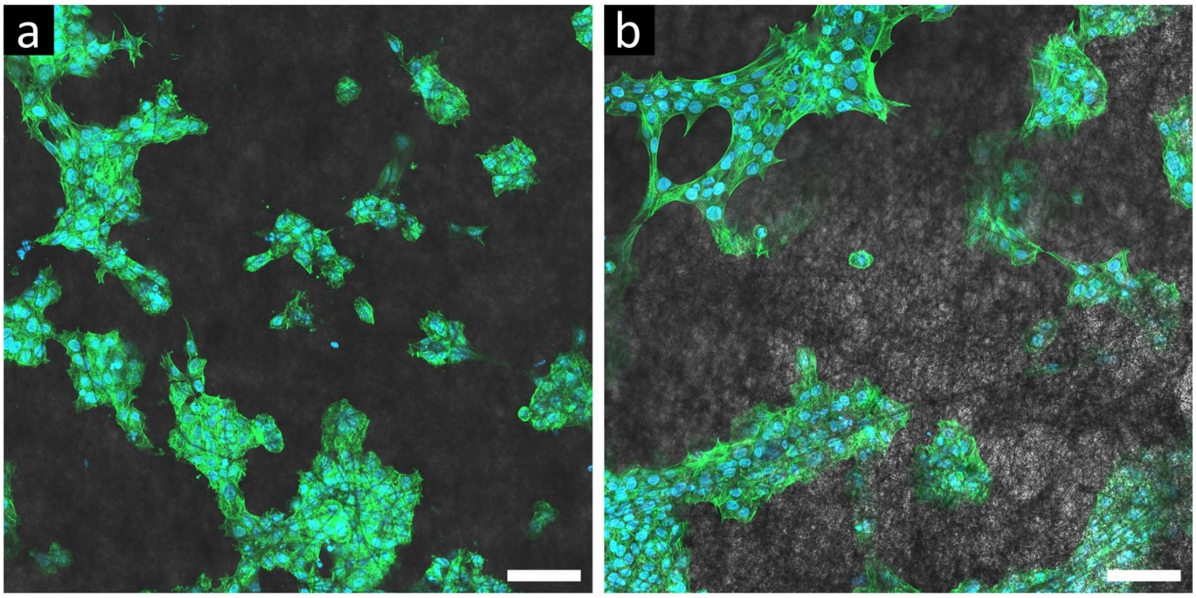

Fig. S2. Cell morphology of cells growing on scaffolds. CLSM images of HaCaT cells on PI fibers **(a)** and on PI+Chol fibers **(b)** after 7 days of culture. The nuclei were stained withDAPI (blue), and the actin filaments with Alexa Fluor 488 Phalloidin (green). Scale bar = 100 µm.
